# Bis(2-amino-4-methyl­pyrimidin-1-ium) hexa­aqua­cobalt(II) disulfate dihydrate

**DOI:** 10.1107/S1600536813002146

**Published:** 2013-01-31

**Authors:** M. Mirzaei, H. Eshtiagh-Hosseini, S. Zarghami, Z. Karrabi, M. Saeedi, J. T. Mague

**Affiliations:** a Department of Chemistry, Ferdowsi University of Mashhad, 917751436 Mashhad, Iran; b Department of Chemistry, Tulane University, New Orleans, LA 70118, USA

## Abstract

In the title hydrated mixed-cation salt, (C_5_H_8_N_3_)_2_[Co(H_2_O)_6_](SO_4_)_2_·2H_2_O, the complete octa­hedral hexa­aqua complex cation is generated by crystallographic inversion symmetry. In the crystal, the components are linked by O—H⋯O and N—H⋯O hydrogen bonds, the latter, involving pyrimidinium cations and sulfate anions, generating *R*_2_^2^(8) loops. These, together with π–π inter­actions between centrosymmetrically related pyrimidinium cations [centroid–centroid separation = 3.5460 (8) Å], lead to the formation of a three-dimensional network.

## Related literature

For a report of the structure of the [Co(H_2_O)_6_]^2+^ ion, see: Shiu *et al.* (2004 ▶). 
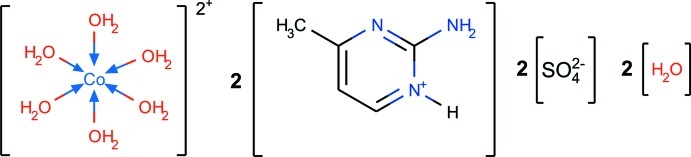


## Experimental

### 

#### Crystal data

(C_5_H_8_N_3_)_2_[Co(H_2_O)_6_](SO_4_)_2_·2H_2_O
*M* *_r_* = 615.49
Triclinic, 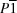

*a* = 6.4116 (6) Å
*b* = 7.7751 (7) Å
*c* = 13.0423 (12) Å
α = 80.136 (1)°
β = 80.413 (1)°
γ = 73.231 (1)°
*V* = 608.57 (10) Å^3^
*Z* = 1
Mo *K*α radiation
μ = 0.96 mm^−1^
*T* = 100 K
0.19 × 0.19 × 0.12 mm

#### Data collection

Bruker SMART APEX CCD diffractometer
Absorption correction: multi-scan (*SADABS*; Sheldrick, 2009 ▶) *T*_min_ = 0.780, *T*_max_ = 0.893
10780 measured reflections
3085 independent reflections
2944 reflections with *I* > 2σ(*I*)
*R*_int_ = 0.025

#### Refinement

*R*[*F* ^2^ > 2σ(*F* ^2^)] = 0.026
*wR*(*F* ^2^) = 0.073
*S* = 1.09
3085 reflections
161 parameters
H-atom parameters constrained
Δρ_max_ = 0.59 e Å^−3^
Δρ_min_ = −0.40 e Å^−3^

### 

Data collection: *APEX2* (Bruker, 2010 ▶); cell refinement: *SAINT* (Bruker, 2009 ▶); data reduction: *SAINT*; program(s) used to solve structure: *SHELXM* (Sheldrick, 2008 ▶); program(s) used to refine structure: *SHELXTL* (Sheldrick, 2008 ▶); molecular graphics: *OLEX2* (Dolomanov *et al.*, 2009 ▶); software used to prepare material for publication: *SHELXTL*.

## Supplementary Material

Crystal structure: contains datablock(s) global, I. DOI: 10.1107/S1600536813002146/hb7029sup1.cif

Structure factors: contains datablock(s) I. DOI: 10.1107/S1600536813002146/hb7029Isup2.hkl

Additional supplementary materials:  crystallographic information; 3D view; checkCIF report

## Figures and Tables

**Table 1:** Selected bond lengths (Å)

Co1—O1	2.0838 (9)
Co1—O2	2.0643 (9)
Co1—O3	2.1140 (10)

**Table 2:** Hydrogen-bond geometry (Å, °)

*D*—H⋯*A*	*D*—H	H⋯*A*	*D*⋯*A*	*D*—H⋯*A*
O1—H1*A*⋯O8	0.84	1.92	2.7562 (14)	171
O1—H1*B*⋯O4	0.84	1.97	2.8050 (13)	178
O2—H2*A*⋯O4^i^	0.84	1.92	2.7533 (14)	173
O2—H2*B*⋯O5	0.84	1.87	2.7077 (13)	174
O3—H3*C*⋯O8^i^	0.84	1.92	2.7508 (13)	170
O3—H3*D*⋯O7^ii^	0.84	1.95	2.7865 (14)	177
N2—H2*N*⋯O6^iii^	0.91	1.81	2.7155 (15)	172
N3—H3*A*⋯O5^iii^	0.91	1.87	2.7776 (15)	175
N3—H3*B*⋯O6^iv^	0.91	1.98	2.8775 (15)	168
O8—H8*A*⋯O7^ii^	0.84	1.98	2.7684 (14)	156
O8—H8*B*⋯O7^v^	0.84	2.02	2.8582 (14)	172

Symmetry codes: (i) 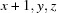; (ii) 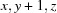; (iii) 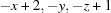; (iv) 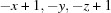; (v) 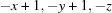.

**Fig. 1.**
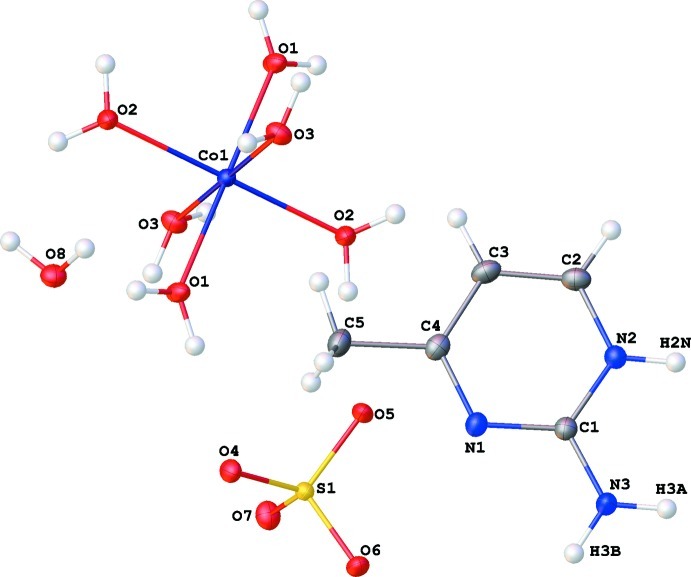
Perspective view of the title compound with 50% probability ellipsoids.

**Fig. 2.**
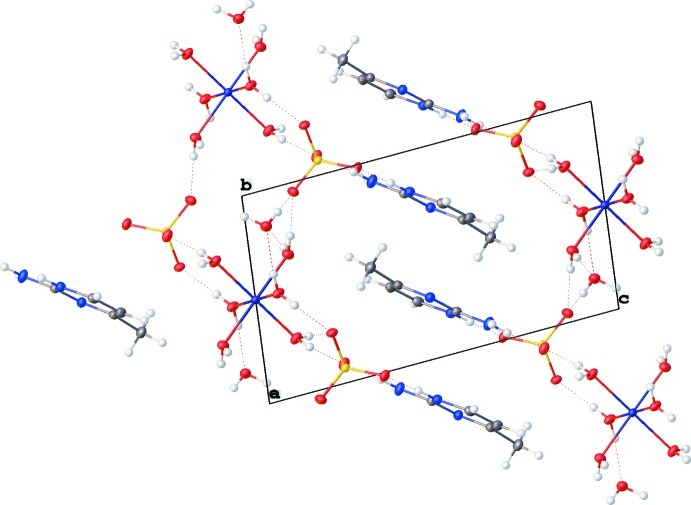
Packing of the title compound viewed down a. Hydrogen bonds are indicated by dotted lines.

**Fig. 3.**
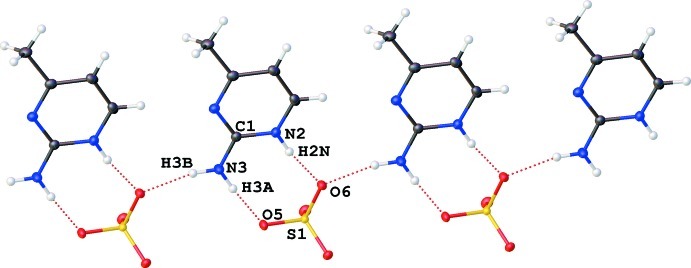
N—H···O hydrogen bonds and the R22(8) synthons (N2, N3, O5 O6).

## supplementary crystallographic information

### Comment

In an attempt to synthesize a cobalt complex of quinoxaline-2,3-dicarboxylic acid by conventional proton transfer processes, cobalt(II) sulfate hexahydrate and the acid were reacted with 2-amino-4-methylpyrimidine. The crystalline product obtained proved to be [Co(H_2_O)_6_][2a-4m-pym]_2_(SO_4_)_2_.2H_2_O (2a-4m-pym = 2-amino-4-methylpyrimidinium) with the cobalt cation having crystallographiclly imposed centrosymmetry (Fig. 1). The Co—O distances range from 2.0643 (9) to 2.1140 (10) A° while the O—Co—O angles range from 90.69 (4) to 93.59 (4)° leading to a somewhat distorted octahedral coordination geometry. The [Co(H_2_O)_6_]^2+^ cations and uncoordinated water molecules form sheets parallel to [110] which are capped on both sides by sulfate ions and held together by O—H···O hydrogen bonds (Fig. 2). N—H···O interactions bind the pyrimidinium cations to the sulfate ions (Fig. 3) with the cations from adjacent sheets intercalating one another (Fig. 2) and stabilized by pairwise π···π (3.546 A°) interactions between them (center pair of cations in Fig. 2). The N2—H2n···O6 and N3—H3a···O5 hydrogen bonding interactions generate *R*_2_^2^(8) synthons.

### Experimental

An aqueous solution of cobalt(II) sulfate hexahydrate (0.4 mmol, 0.8 mg) in distilled water (5 ml) was added to an aqueous solution of quinoxaline-2,3-dicarboxylic acid (0.11 mmol, 25 mg) and 2-amino-4-methyl pyrimidine (0.24 mmol, 26 mg). The mixture refluxed for 5 hrs at 75°C. Orange blocks were obtained by slow evaporation of the reaction mixture at room temperature.

### Crystal data

**Table:**

(C_5_H_8_N_3_)_2_[Co(H_2_O)_6_](SO_4_)_2_·2H_2_O	*Z* = 1
*M**_r_* = 615.49	*F*(000) = 321
Triclinic, *P*1	*D*_x_ = 1.679 Mg m^−^^3^
Hall symbol: -P 1	Mo *K*α radiation, λ = 0.71073 Å
*a* = 6.4116 (6) Å	Cell parameters from 8977 reflections
*b* = 7.7751 (7) Å	θ = 2.8–29.2°
*c* = 13.0423 (12) Å	µ = 0.96 mm^−^^1^
α = 80.136 (1)°	*T* = 100 K
β = 80.413 (1)°	Block, orange
γ = 73.231 (1)°	0.19 × 0.19 × 0.12 mm
*V* = 608.57 (10) Å^3^	

### Data collection

**Table:**

Bruker SMART APEX CCD diffractometer	3085 independent reflections
Radiation source: fine-focus sealed tube	2944 reflections with *I* > 2σ(*I*)
Graphite monochromator	*R*_int_ = 0.025
φ and ω scans	θ_max_ = 29.2°, θ_min_ = 2.8°
Absorption correction: multi-scan (*SADABS*; Sheldrick, 2009)	*h* = −8→8
*T*_min_ = 0.780, *T*_max_ = 0.893	*k* = −10→10
10780 measured reflections	*l* = −17→17

### Refinement

**Table:**

Refinement on *F*^2^	Primary atom site location: structure-invariant direct methods
Least-squares matrix: full	Secondary atom site location: difference Fourier map
*R*[*F*^2^ > 2σ(*F*^2^)] = 0.026	Hydrogen site location: inferred from neighbouring sites
*wR*(*F*^2^) = 0.073	H-atom parameters constrained
*S* = 1.09	*w* = 1/[σ^2^(*F*_o_^2^) + (0.0386*P*)^2^ + 0.2936*P*] where *P* = (*F*_o_^2^ + 2*F*_c_^2^)/3
3085 reflections	(Δ/σ)_max_ = 0.001
161 parameters	Δρ_max_ = 0.59 e Å^−^^3^
0 restraints	Δρ_min_ = −0.40 e Å^−^^3^

### Special details

**Table:**

Experimental. The diffraction data were obtained from 3 sets of 400 frames, each of width 0.5 °. in omega, collected at phi = 0.00, 90.00 and 180.00 °. and 2 sets of 800 frames, each of width 0.45 ° in phi, collected at omega = -30.00 and 210.00 °. The scan time was 10 sec/frame.
Geometry. All e.s.d.'s (except the e.s.d. in the dihedral angle between two l.s. planes) are estimated using the full covariance matrix. The cell e.s.d.'s are taken into account individually in the estimation of e.s.d.'s in distances, angles and torsion angles; correlations between e.s.d.'s in cell parameters are only used when they are defined by crystal symmetry. An approximate (isotropic) treatment of cell e.s.d.'s is used for estimating e.s.d.'s involving l.s. planes.
Refinement. Refinement of *F*^2^ against ALL reflections. The weighted *R*-factor *wR* and goodness of fit *S* are based on *F*^2^, conventional *R*-factors *R* are based on *F*, with *F* set to zero for negative *F*^2^. The threshold expression of *F*^2^ > σ(*F*^2^) is used only for calculating *R*-factors(gt) *etc*. and is not relevant to the choice of reflections for refinement. *R*-factors based on *F*^2^ are statistically about twice as large as those based on *F*, and *R*- factors based on ALL data will be even larger. H-atoms attached to carbon were placed in calculated positions (C—H = 0.95 - 0.98 Å) while those attached to nitrogen and oxygen were placed in locations derived from a difference map. All were included as riding contributions with isotropic displacement parameters 1.2 - 1.5 times those of the attached atoms.

### Fractional atomic coordinates and isotropic or equivalent isotropic displacement parameters (Å^2^)

**Table:**

	*x*	*y*	*z*	*U*_iso_*/*U*_eq_	
Co1	1.0000	0.5000	0.0000	0.00955 (8)	
O1	0.68016 (15)	0.50215 (13)	0.06589 (8)	0.01452 (19)	
H1A	0.5761	0.5965	0.0592	0.017*	
H1B	0.6253	0.4255	0.1061	0.017*	
O2	1.13866 (16)	0.27066 (13)	0.09714 (8)	0.0152 (2)	
H2A	1.2384	0.2644	0.1331	0.018*	
H2B	1.0513	0.2134	0.1317	0.018*	
O3	1.01700 (15)	0.67088 (13)	0.10641 (8)	0.01429 (19)	
H3C	1.1304	0.7080	0.0967	0.017*	
H3D	0.9025	0.7538	0.1193	0.017*	
N1	0.77618 (19)	0.29129 (15)	0.48276 (9)	0.0141 (2)	
N2	1.13318 (19)	0.19273 (15)	0.53464 (9)	0.0136 (2)	
H2N	1.2214	0.1283	0.5837	0.016*	
N3	0.8459 (2)	0.09569 (16)	0.63508 (9)	0.0169 (2)	
H3A	0.9370	0.0291	0.6825	0.020*	
H3B	0.7047	0.0884	0.6443	0.020*	
C1	0.9176 (2)	0.19330 (17)	0.55062 (10)	0.0126 (2)	
C2	1.2141 (2)	0.28771 (18)	0.44771 (11)	0.0159 (3)	
H2	1.3655	0.2840	0.4361	0.019*	
C3	1.0775 (2)	0.38846 (18)	0.37710 (11)	0.0164 (3)	
H3	1.1307	0.4555	0.3155	0.020*	
C4	0.8539 (2)	0.38965 (18)	0.39882 (10)	0.0143 (2)	
C5	0.6904 (2)	0.50542 (19)	0.32901 (11)	0.0184 (3)	
H5A	0.6319	0.4281	0.2960	0.028*	
H5B	0.7619	0.5807	0.2746	0.028*	
H5C	0.5701	0.5834	0.3706	0.028*	
S1	0.64309 (5)	0.07057 (4)	0.22320 (2)	0.00995 (8)	
O4	0.48922 (15)	0.25043 (12)	0.19945 (8)	0.01356 (19)	
O5	0.86583 (15)	0.09036 (13)	0.22097 (8)	0.0153 (2)	
O6	0.57348 (16)	−0.01584 (13)	0.32903 (8)	0.0148 (2)	
O7	0.64425 (16)	−0.04477 (13)	0.14342 (8)	0.0164 (2)	
O8	0.36083 (16)	0.82758 (13)	0.05699 (8)	0.01547 (19)	
H8A	0.4119	0.8803	0.0939	0.019*	
H8B	0.3513	0.8994	0.0009	0.019*	

### Atomic displacement parameters (Å^2^)

**Table:**

	*U*^11^	*U*^22^	*U*^33^	*U*^12^	*U*^13^	*U*^23^
Co1	0.00874 (12)	0.00915 (12)	0.01048 (13)	−0.00265 (8)	−0.00139 (9)	0.00014 (8)
O1	0.0100 (4)	0.0120 (4)	0.0190 (5)	−0.0025 (3)	−0.0001 (4)	0.0023 (3)
O2	0.0128 (4)	0.0151 (4)	0.0179 (5)	−0.0061 (4)	−0.0050 (4)	0.0048 (4)
O3	0.0107 (4)	0.0149 (4)	0.0178 (5)	−0.0036 (3)	−0.0001 (4)	−0.0047 (4)
N1	0.0149 (5)	0.0136 (5)	0.0133 (5)	−0.0032 (4)	−0.0032 (4)	−0.0005 (4)
N2	0.0125 (5)	0.0137 (5)	0.0144 (5)	−0.0030 (4)	−0.0024 (4)	−0.0013 (4)
N3	0.0140 (5)	0.0206 (6)	0.0157 (6)	−0.0065 (4)	−0.0047 (4)	0.0053 (4)
C1	0.0136 (6)	0.0114 (6)	0.0130 (6)	−0.0029 (4)	−0.0018 (5)	−0.0025 (4)
C2	0.0150 (6)	0.0150 (6)	0.0176 (6)	−0.0052 (5)	0.0031 (5)	−0.0045 (5)
C3	0.0195 (7)	0.0142 (6)	0.0141 (6)	−0.0053 (5)	0.0031 (5)	−0.0022 (5)
C4	0.0187 (6)	0.0119 (6)	0.0119 (6)	−0.0028 (5)	−0.0015 (5)	−0.0033 (4)
C5	0.0222 (7)	0.0171 (6)	0.0155 (6)	−0.0049 (5)	−0.0061 (5)	0.0017 (5)
S1	0.00895 (15)	0.01018 (15)	0.01081 (15)	−0.00313 (11)	−0.00176 (11)	−0.00010 (11)
O4	0.0121 (4)	0.0105 (4)	0.0172 (5)	−0.0015 (3)	−0.0033 (4)	−0.0003 (3)
O5	0.0103 (4)	0.0192 (5)	0.0165 (5)	−0.0067 (4)	−0.0035 (4)	0.0039 (4)
O6	0.0126 (4)	0.0167 (5)	0.0139 (5)	−0.0054 (4)	−0.0010 (4)	0.0033 (4)
O7	0.0178 (5)	0.0149 (5)	0.0171 (5)	−0.0022 (4)	−0.0044 (4)	−0.0054 (4)
O8	0.0165 (5)	0.0142 (4)	0.0172 (5)	−0.0062 (4)	−0.0021 (4)	−0.0024 (4)

### Geometric parameters (Å, º)

**Table:**

Co1—O1	2.0838 (9)	N3—H3A	0.9099
Co1—O1^i^	2.0838 (9)	N3—H3B	0.9100
Co1—O2	2.0643 (9)	N3—C1	1.3216 (17)
Co1—O2^i^	2.0643 (9)	C2—H2	0.9500
Co1—O3^i^	2.1140 (10)	C2—C3	1.362 (2)
Co1—O3	2.1140 (10)	C3—H3	0.9500
O1—H1A	0.8400	C3—C4	1.412 (2)
O1—H1B	0.8400	C4—C5	1.4935 (19)
O2—H2A	0.8400	C5—H5A	0.9800
O2—H2B	0.8400	C5—H5B	0.9800
O3—H3C	0.8400	C5—H5C	0.9800
O3—H3D	0.8400	S1—O4	1.4772 (10)
N1—C1	1.3503 (17)	S1—O5	1.4749 (9)
N1—C4	1.3352 (17)	S1—O6	1.4851 (10)
N2—H2N	0.9101	S1—O7	1.4834 (10)
N2—C1	1.3621 (17)	O8—H8A	0.8400
N2—C2	1.3558 (17)	O8—H8B	0.8400
			
O1^i^—Co1—O1	180.0	H3A—N3—H3B	118.7
O1^i^—Co1—O3	89.31 (4)	C1—N3—H3A	121.5
O1—Co1—O3	90.69 (4)	C1—N3—H3B	119.7
O1^i^—Co1—O3^i^	90.69 (4)	N1—C1—N2	121.68 (12)
O1—Co1—O3^i^	89.31 (4)	N3—C1—N1	119.51 (12)
O2^i^—Co1—O1^i^	93.59 (4)	N3—C1—N2	118.82 (12)
O2—Co1—O1	93.59 (4)	N2—C2—H2	120.1
O2^i^—Co1—O1	86.41 (4)	N2—C2—C3	119.73 (13)
O2—Co1—O1^i^	86.41 (4)	C3—C2—H2	120.1
O2—Co1—O2^i^	180.0	C2—C3—H3	121.2
O2^i^—Co1—O3	88.21 (4)	C2—C3—C4	117.61 (12)
O2^i^—Co1—O3^i^	91.79 (4)	C4—C3—H3	121.2
O2—Co1—O3^i^	88.21 (4)	N1—C4—C3	122.45 (12)
O2—Co1—O3	91.79 (4)	N1—C4—C5	116.47 (12)
O3—Co1—O3^i^	180.0	C3—C4—C5	121.07 (12)
Co1—O1—H1A	121.3	C4—C5—H5A	109.5
Co1—O1—H1B	133.1	C4—C5—H5B	109.5
H1A—O1—H1B	105.3	C4—C5—H5C	109.5
Co1—O2—H2A	123.6	H5A—C5—H5B	109.5
Co1—O2—H2B	115.8	H5A—C5—H5C	109.5
H2A—O2—H2B	109.5	H5B—C5—H5C	109.5
Co1—O3—H3C	116.1	O4—S1—O6	110.08 (6)
Co1—O3—H3D	115.8	O4—S1—O7	108.96 (6)
H3C—O3—H3D	112.0	O5—S1—O4	109.75 (6)
C4—N1—C1	117.86 (12)	O5—S1—O6	108.84 (6)
C1—N2—H2N	118.7	O5—S1—O7	109.68 (6)
C2—N2—H2N	120.7	O7—S1—O6	109.52 (6)
C2—N2—C1	120.59 (12)	H8A—O8—H8B	102.1
			
N2—C2—C3—C4	0.4 (2)	C2—N2—C1—N3	178.45 (12)
C1—N1—C4—C3	2.59 (19)	C2—C3—C4—N1	−2.6 (2)
C1—N1—C4—C5	−176.26 (12)	C2—C3—C4—C5	176.18 (12)
C1—N2—C2—C3	1.75 (19)	C4—N1—C1—N2	−0.37 (19)
C2—N2—C1—N1	−1.82 (19)	C4—N1—C1—N3	179.37 (12)

Symmetry code: (i) −*x*+2, −*y*+1, −*z*.

### Hydrogen-bond geometry (Å, º)

**Table:**

*D*—H···*A*	*D*—H	H···*A*	*D*···*A*	*D*—H···*A*
O1—H1*A*···O8	0.84	1.92	2.7562 (14)	171
O1—H1*B*···O4	0.84	1.97	2.8050 (13)	178
O2—H2*A*···O4^ii^	0.84	1.92	2.7533 (14)	173
O2—H2*B*···O5	0.84	1.87	2.7077 (13)	174
O3—H3*C*···O8^ii^	0.84	1.92	2.7508 (13)	170
O3—H3*D*···O7^iii^	0.84	1.95	2.7865 (14)	177
N2—H2*N*···O6^iv^	0.91	1.81	2.7155 (15)	172
N3—H3*A*···O5^iv^	0.91	1.87	2.7776 (15)	175
N3—H3*B*···O6^v^	0.91	1.98	2.8775 (15)	168
O8—H8*A*···O7^iii^	0.84	1.98	2.7684 (14)	156
O8—H8*B*···O7^vi^	0.84	2.02	2.8582 (14)	172

Symmetry codes: (ii) *x*+1, *y*, *z*; (iii) *x*, *y*+1, *z*; (iv) −*x*+2, −*y*, −*z*+1; (v) −*x*+1, −*y*, −*z*+1; (vi) −*x*+1, −*y*+1, −*z*.
